# Acute Stress Facilitates LTD Induction at Glutamatergic Synapses in the Hippocampal CA1 Region by Activating μ-Opioid Receptors on GABAergic Neurons

**DOI:** 10.3389/fnins.2019.00071

**Published:** 2019-02-08

**Authors:** Ka-Min Fan, Li-Juan Qiu, Ning Ma, Yi-Nan Du, Zhao-Qiang Qian, Chun-Ling Wei, Jing Han, Wei Ren, Mei-Mei Shi, Zhi-Qiang Liu

**Affiliations:** MOE Key Laboratory of Modern Teaching Technology, Center for Teacher Professional Ability Development, Shaanxi Normal University, Xi’an, China

**Keywords:** synaptic plasticity, hippocampus, endogenous opioid system, acute stress, inhibitory transmission, μ-opioid receptors

## Abstract

Acute stress impairs recall memory through the facilitation of long-term depression (LTD) of hippocampal synaptic transmission. The endogenous opioid system (EOS) plays essential roles in stress-related emotional and physiological responses. Specifically, behavioral studies have shown that the impairment of memory retrieval induced by stressful events involves the activation of opioid receptors. However, it is unclear whether signaling mediated by μ-opioid receptors (μRs), one of the three major opioid receptors, participates in acute stress-related hippocampal LTD facilitation. Here, we examined the effects of a single elevated platform (EP) stress exposure on excitatory synaptic transmission and plasticity at the Schaffer collateral-commissural (SC) to CA1 synapses by recording electrically evoked field excitatory postsynaptic potentials and population spikes of hippocampal pyramidal neurons in anesthetized adult mice. EP stress exposure attenuated GABAergic feedforward and feedback inhibition of CA1 pyramidal neurons and facilitated low-frequency stimulation (LFS)-induced long-term depression (LTD) at SC-CA1 glutamatergic synapses. These effects were reproduced by exogenously activating μRs in unstressed mice. The specific deletion of μRs on GABAergic neurons (μR_GABA_) not only prevented the EP stress-induced memory impairment but also reversed the EP stress-induced attenuation of GABAergic inhibition and facilitation of LFS-LTD. Our results suggest that acute stress endogenously activates μR_GABA_ to attenuate hippocampal GABAergic signaling, thereby facilitating LTD induction at excitatory synapses and eliciting memory impairments.

## Introduction

Acute stress markedly influences cognitive functions (de Quervain et al., 1998; Kuhlmann et al., 2005; Wong et al., 2007) through cellular mechanisms that alter excitatory synaptic plasticity in relevant brain regions (Kim et al., 1996; Joels et al., 2006). Specifically, in the CA1 region of the hippocampus, acute stress facilitates the induction of N-methyl-D-aspartate receptor (NMDAR)-dependent long-term depression (LTD), which is tentatively linked to acute stress-induced impairment of spatial memory retrieval (Xu et al., 1997; Wong et al., 2007). Furthermore, artificial facilitation of LTD induction in the hippocampal CA1 of adult rats using glutamate transporter inhibitors reproduces the effects of acute stress on hippocampus-dependent spatial memory retrieval (Wong et al., 2007). This finding suggests that LTD plays a critical role in the behavioral responses to acute stress. However, the distinct mechanisms underlying stress facilitation of LTD are not well understood.

Previous studies have revealed that LTD is involved in spatial learning and memory (Brigman et al., 2010; Ge et al., 2010) and is a possible mechanism for memory “deletion” or “forgetting” (Collingridge et al., 2010). In experiments, the expression of hippocampal LTD is generally dependent on the stimulus pattern (Buschler et al., 2012) and on the developmental stage of the animals (Wagner and Alger, 1995). Classical low-frequency stimulation (LFS, usually 1–5 Hz, 900 pulses) consistently induces NMDAR-dependent LTD at synapses of Schaffer collateral/commissural (SC) fibers on CA1 neurons in young animals (18–22 day-old) (Wagner and Alger, 1995). In contrast, a similar LFS protocol elicits only short-term depression (Buschler et al., 2012) but not LTD in adult (5–10 week-old) animals both *in vivo* (Xu et al., 1998; Wong et al., 2007; Buschler et al., 2012) and *in vitro* (Kim et al., 1996; Otani and Connor, 1998). Thus, the facilitation of LTD induction in adult animals under certain conditions, such as acute stress exposure, suggests that such conditions might alter the induction threshold of hippocampal LTD.

Hippocampal GABAergic interneurons play a central role in modulating the synaptic plasticity of excitatory synapses (Wigstrom and Gustafsson, 1983; Kerr and Abraham, 1995; Nusser and Mody, 2002). In particular, postsynaptic GABA_A_ receptors regulate the threshold of LTD induction by shunting (as phasic inhibition) and hyperpolarizing (as tonic inhibition) pyramidal neurons (Otani and Connor, 1998; Nusser and Mody, 2002). In adult animals, the strong effects of GABAergic inhibition on NMDAR activation during the LFS episode effectively restrict the induction of NMDAR-dependent LTD, whereas the removal of GABAergic inhibition by blockade of GABA_A_ receptors facilitates LTD induction (Wagner and Alger, 1995; Otani and Connor, 1998). Therefore, the reduction of the LTD threshold by acute stress suggests that an attenuation of GABAergic inhibition may be involved during stress. Indeed, previous studies have revealed that bath application of corticosteroid to hippocampal slices depressed GABAergic transmission in the CA1 region (Maggio and Segal, 2009).

The endogenous opioid system (EOS) has long been implicated in the stress response. The enhanced release of endogenous opioid peptides during stress can either attenuate or aggravate stress responses depending on the specific opioid receptor that is activated (Valentino and Van Bockstaele, 2008). Moreover, the EOS participates in stress-induced memory impairments, as supported by several lines of evidence: blockade of opioid receptors by naloxone can reverse the effect of acute restraint stress to impair the retrieval of long-term memory in the inhibitory avoidance task (Rashidy-Pour et al., 2004); the corticosterone-induced impairment of memory retrieval in the Morris water maze (MWM) task was blocked by intra-hippocampal infusions of naltrexone (Sajadi et al., 2007); and the application of naloxone can also inhibit the impairment of recognition memory retrieval caused by forced swimming stress in a novel object recognition task (Liu et al., 2016). Importantly, our unpublished data show that μ-opioid receptors (μRs) in the hippocampus are endogenously activated by increased enkephalin (one of the major endogenous opioid peptide neurotransmitters) during acute elevated platform (EP) stress. These receptors then trigger the EP stress-induced spatial reference memory impairment in the MWM task (Cao et al., 2015).

Hippocampal μRs are predominantly located at the axons, terminals, dendrites, and somata of GABAergic inhibitory interneurons (Drake and Milner, 2002). As a vital modulator of GABAergic signaling, exogenously activated μRs can disinhibit pyramidal neurons by inhibiting the firing rate and neurotransmitter release of GABAergic interneurons (Drake and Milner, 2002), therefore altering the excitability of CA1 pyramidal neurons and synaptic plasticity in the hippocampus (Dacher and Nugent, 2011). Considering the critical role of the EOS on both memory processing and stress responses (Valentino and Van Bockstaele, 2008; Shields et al., 2017), we hypothesize that endogenous activation of μRs by acute stress leads to modifications of hippocampal synaptic transmission and plasticity, which may contribute to stress-induced memory impairment. In the present study, we recorded evoked field excitatory postsynaptic potentials (fEPSPs) and population spikes (PSs) in pyramidal neurons of anesthetized adult mice, in combination with the MWM task. These studies allowed us to examine the effect of μRs on changes in synaptic transmission and plasticity in the hippocampal CA1 area following a single exposure to EP stress, as well as the contribution of these receptors to stress-induced memory impairment.

## Materials and Methods

### Animals

All experiments involving animals were performed in accordance with the Chinese Council on Animal Care and were approved by the Animal Care Committee of Shaanxi Normal University. Adult male C57BL/6J (from the Model Animal Research Center of Nanjing University, China) and mutant mice (from Beijing Biocytogen, China) weighing 23–26 g were used for all experiments. All animals were housed in groups of 4 in individually ventilated cages (16 × 24 × 12 cm) and maintained at 22 ± 2 °C and 55 ± 5% relative humidity on a 12/12 h light/dark cycle with lights on at 8:00 a.m. Food and water were available *ad libitum*. Behavioral experiments were carried out between 9:00 a.m. and 11:00 a.m.

**FIGURE 1:**
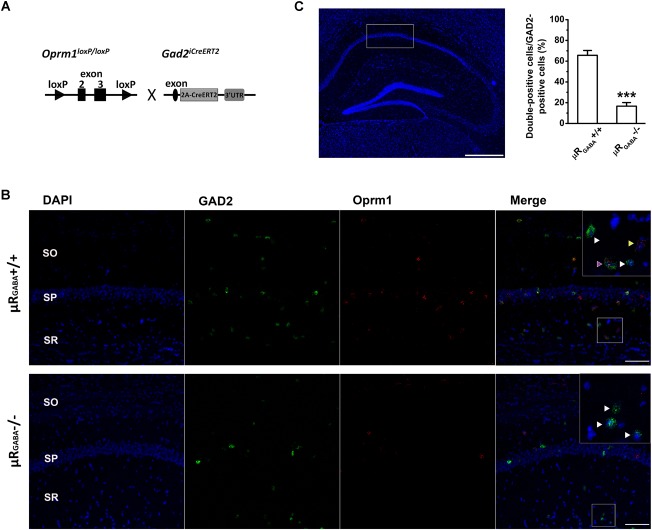
Generation of conditional knockout mice specifically lacking μRs on GABAergic neurons (μR_GABA_-/-). **(A)** A schematic diagram shows that *Oprm1^flox/flox^* mice are crossed with *Gad2^iCreERT2+/+^* mice. Exons 2–3 of *Oprm1* gene are excised in GAD2 positive neurons expressing Cre recombinase. **(B)** Representative images of *in situ* hybridization for *Oprm1* mRNA and GAD2 mRNA in the hippocampal CA1 areas in μR_GABA_+/+ (upper panel) and μR_GABA_-/- (lower panel) mice. The nucleus is stained in blue (DAPI), GAD2 mRNA in green, and *Oprm1* mRNA in red. The insets are higher-magnification images of the fields indicated by the white boxes. The purple arrow-head indicates a double-labeled cell with *Oprm1* mRNA and GAD2 mRNA; the yellow arrow-heads represent μR mRNA localization in GAD2-negative cells; and the white arrow heads represent GAD2-positive cells without μR mRNA. Compared to littermate controls (μR_GABA_+/+), the μR_GABA_-/- mice display an absence of μR mRNA in most GAD2-positive but not GAD2-negative cells. SO, stratum oriens; SP, stratum pyramidale; SR, stratum radiatum. Scale bar = 100 μm. **(C)** Quantitative analysis of the percentige of double (*Oprm1* and GAD2) positive neurons against the GAD2-positive neurons. ^∗∗∗^*p* < 0.001, verse μR_GABA_+/+, unpaired *t*-test (*n* = 4 per group).

A mutant mouse line specifically lacking μRs on GABAergic neurons (μR_GABA_) was generated by crossing *Oprm1* floxed mice bearing conditional alleles of the *Oprm1* gene containing loxP sites flanking exons 2 and 3 (Weibel et al., 2013) with specific *Gad2^iCreERT2^* mice (Zhong et al., 2017; Figure 1A). The adult *Oprm1^flox/flox^:Gad2^iCreERT2+/+^* mice were treated with tamoxifen (Sigma; dissolved in corn oil; 2 mg/day, i.p.) for 7 consecutive days to induce the deletion of μR (μR_GABA_-/-) and then used for experiments 2 weeks after the last injection. The littermate *Oprm1^flox/flox^:Gad2^iCreERT2^*^-^*^/^*^-^ mice (μR_GABA_+/+) treated with the same tamoxifen regimen were used as controls for the μR_GABA_-/- mice. The deletion of the μR protein in the mutant mice was verified using fluorescent *in situ* hybridization (Figure 1B,C).

### Behavioral Stress

The EP stress protocol was conducted as described previously (Wong et al., 2007). Briefly, each mouse was stressed on an elevated circular Plexiglas platform (1.3 m high, 8 cm in diameter) in a quiet and brightly lit room for 50 min. While on the platform, the stress behaviors of the animals (such as increased urination and/or defecation, immobility, piloerection, etc.) were observed. Immediately after stress, the mice were placed in a water maze for administration of a memory retrieval test or anesthetized for electrophysiological recording.

### MWM Task

The spatial reference memory was evaluated using a modified MWM task as described previously (Brigman et al., 2010). Briefly, the apparatus was a circular pool 100 cm in diameter filled with 21 ± 1°C water (depth of 20 cm) and made opaque with non-toxic white tempera paint powder. A circular platform (10 cm in diameter) was hidden 1.0 cm below the water surface in the center (30 cm from the edge of the pool) of 1 (target) of 4 imaginary quadrants throughout training. Visual marks of different colors and dimensions were positioned around the pool as extra-maze cues. For the hidden platform task, mice were placed at 1 of 4 pseudo-randomly chosen start positions that were equally distributed around the perimeter of the maze and allowed to search for the hidden platform for 60 s. Once on the platform, mice were allowed to remain there for 20 s, and then they were placed in a holding cage for 10 min until the next trial. Mice that failed to find the hidden platform within 60 s were manually guided there. Mice were subjected to 4 trials per day over 4 consecutive days. Twenty-four hours after the last training session, the mice were placed in the pool for the probe test at a position equidistant from the target quadrant and in the opposite quadrant. The probe trial consisted of a 60 s free-swim period with the platform removed. Swimming paths for all trials were monitored using the Ethovision video tracking system (Noldus, Leesburg, VA, United States). The escape latency (latency to find the platform) during the training session, the average swimming speed and the time spent in each of the four quadrants during the probe trial were measured.

### Fluorescent *in situ* Hybridization With RNAscope

The deletion of μRs in mutant mice was verified using an *in situ* hybridization assay (Figure 1B,C). Briefly, mice were anesthetized with 25% urethane (Sigma, 1 g/kg, i.p.) and then perfused with 0.9% normal saline. The brain was snap-frozen over dry ice and embedded in OCT (Sakura Finetek, CA, United States, catalog number: 4583). Fresh frozen sections (16 μm) were cut coronally through the hippocampal formation using a freezing microtome (CM1950, Leica Microsciences, Germany) and thaw-mounted onto Superfrost Plus Microscope Slides (Thermo Fisher Scientific, catalog number: 12-550-15). Slides were fixed in 4% PFA for 60 min at 4°C before being dehydrated using a graded ethanol series at room temperature for 5 min each (50, 70, 100, and 100%) and then air-dried. The sections were incubated with H_2_O_2_ for 10 min and subsequently pretreated with protease IV for 15 min. The probes for *Oprm1* (sixteen synthetic oligonucleotides complementary to the nucleotide sequence 590–1458 of *Oprm1*) and Gad2 (catalog number: 439371) were provided by Advanced Cell Diagnostics and conjugated to Atto 550 and Atto 647, respectively. The procedure for *in situ* detection was performed using an RNAscope Multiplex Fluorescent Reagent Kit v2 (Advanced Cell Diagnostics, CA, United States. catalog number: 323100) according to the manufacturer’s instructions for fresh frozen tissue. The HybEZTM oven (Advanced Cell Diagnostics, CA, United States) was used in the heating steps, and the slides were mounted using Prolong Gold Antifade (Thermo Fisher Scientific, Molecular Probes, catalog number: P10144). Confocal images were assessed using a laser scanning microscope (Leica, TCS SP5) and positive labeling was counted.

### Field Potential Recording of Anesthetized Mice

Mice were anesthetized with 25% urethane (Sigma, 1 g/kg, i.p.) and mounted on a stereotaxic frame (Stolting, Wood Dale, IL, United States). The body temperature of the mice was maintained at 36.5°C using an electric heating pad. Two small holes were drilled into the skull. A bipolar stimulating electrode (Kopf, Tujunga, CA, United States) was inserted in the contralateral SC pathway of the right dorsal hippocampus (1.5 mm posterior to bregma, 2.5 mm lateral to midline, 1.0∼1.5 mm below the surface of the cortex). A recording micropipette (tip diameter 2∼3 μm, 2∼5 MΩ resistance when filled with 2 M NaCl and 2% pontamine sky blue) was inserted in the CA1 region pyramidal cell layer (2.1 mm posterior to bregma, 1.5 mm lateral to midline, DV ∼1.1 mm from brain surface). The evoked PSs and fEPSPs were separately collected in different animals, while the recording electrode was placed in the pyramidal cell layer (1.1 ± 0.1 mm below the surface of the cortex) for PSs or in the stratum radiatum layer (1.3 ± 0.2 mm below the surface of the cortex) for fEPSPs. Single-pulse stimulation (biphasic square waves of 0.2 ms duration per half-wave) using a stimulator (S88X, Grass Technologies, West Warwick, RI, United States) was delivered at 0.033 Hz, and the response was amplified using an amplifier (Axoclamp 900A, Axon Instruments, CA, United States). Data acquisition and analysis were performed using a digitizer (Digidata 1440A, Axon Instruments) and Clampfit 10.2 analysis software (Molecular Devices), respectively. The test stimulus intensity used to produce a specific fEPSP or PS amplitude was adjusted to 30∼50% of the maximal value. The PS amplitude (Figure 3A) was quantified by measuring the values from the lowest point of the PS to the pre-PS peak (A1) and to the post-PS peak (A2) and calculated with the following formula: (A1 + A2)/2 × 100%. The input/output curves of the fEPSPs were constructed by varying the stimulus intensity (10–650 mA) and measuring the peak fEPSP amplitude. The paired-pulse ratio (PPR) of the EPSPs or PSs was calculated by determining the ratio of the peak amplitude values of fEPSP2/fEPSP1 or PS2/PS1 induced by double-pulse stimulation at each inter-pulse interval (IPI: 50, 100, and 200 ms for fEPSP, and 25 ms for PS). The average of 10 fEPSPs or PSs was collected at each IPI point. LTD of fEPSPs was induced using LFS (900 pulses at 3 Hz) after obtaining a stable baseline for at least 10 min. The level of LTD was reported as a comparison of the average fEPSP amplitude for a 5 min period immediately before LTD induction with that of a period from 41 to 50 min after LFS. At the end of the study, the mouse brains were removed and sliced to verify of the stimulating/recording electrode localization. The data were discarded if incorrect electrode localization was detected.

### Chemicals and Reagents

Morphine hydrochloride (Qinghai Pharmaceutical Co.), bicuculline methiodide (Sigma) and naloxone hydrochloride dihydrate (Sigma) were dissolved in saline (0.9% NaCl) and administered intraperitoneally (i.p.) to anesthetized mice.

### Statistical Analyses

All data are presented as the mean ± S.E.M (in some cases, mean ± S.E.M%) and were analyzed using SPSS 18.0 software. The Student’s *t*-test, one-way analysis of variance (ANOVA), two-way ANOVA, or repeated-measures (R-M) ANOVA was used for individual comparisons as described in the results section. The ANOVAs were followed by the Bonferroni *post hoc* test or the Student’s *t*-test. Statistical significance was set at *p* < 0.05.

## Results

**FIGURE 2:**
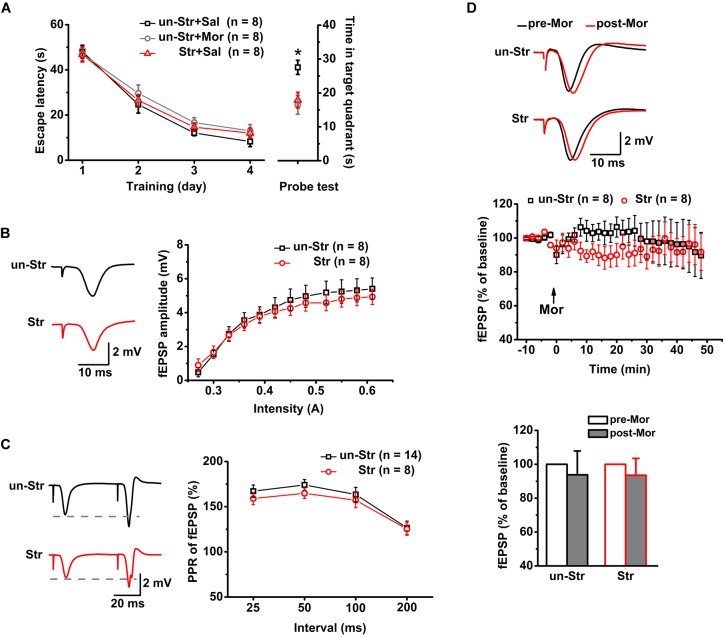
The effects of EP stress on memory retrieval and excitatory synaptic transmission in the CA1 region in wildtype mice. **(A)** Impairment of memory retrieval induced by EP stress or morphine injection before the probe test. All groups exhibited a similar escape latency during the MWM training days [group *F*_(2_, _21)_ = 1.12, *p* = 0.347; training day *F*_(3_, _63)_ = 121.07, *p* = 0.000; group × training day *F*_(6_, _63)_ = 0.37, *p* = 0.858; R-M ANOVA). Time spent in target quadrant of test trial *F*_(2_, _21)_ = 6.30, *p* = 0.007, one-way ANOVA; ^∗^*p* < 0.05 versus Sal + un-Str or Mor + un-Str group, Bonferroni *post hoc* test. **(B)** Stress does not alter the input/output curve of fEPSP amplitude. Treatment (stressed or unstressed) *F*_(1_, _14)_ = 0.45, *p* = 0.512; intensity *F*_(11_, _154)_ = 57.31, *p* = 0.000; treatment × intensity *F*_(11_, _154)_ = 1.15, *p* = 0.320; R-M ANOVA. **(C)** Stress does not change the PPR of fEPSPs at various IPIs. Treatment *F*_(1_, _20)_ = 0.50, *p* = 0.488; interval *F*_(3_, _60)_ = 29.13, *p* = 0.000; treatment × interval *F*_(3_, _60)_ = 0.87, *p* = 0.232; R-M ANOVA. **(D)** Morphine injection (15 mg/kg, i.p.) does not affect the fEPSP amplitude of unstressed and stressed mice. The bottom panel shows the histograms of fEPSP amplitude measurements (averaged from a 5 min period either before or after a 45 min morphine application) relative to baseline. Treatment (with or without morphine) *F*_(1_, _14)_ = 0.54, *p* = 0.475; group (with or without stress) *F*_(1_, _14)_ = 0.00, *p* = 0.989; treatment × group *F*_(1_, _14)_ = 0.00, *p* = 0.989; R-M ANOVA. un-Str, unstressed; Str, stressed; Sal, saline; Mor, morphine.

Twenty-four mice were trained in the MWM task. Subsequently, the trained mice were randomly divided into the following groups: unstressed with saline injection, unstressed with morphine injection, and stressed with saline injection (*n* = 8 for each group). Memory retrieval was evaluated 24 h after the last training session. Compared to the unstressed group that received the saline treatment, the unstressed group that received morphine (15 mg/kg, i.p.), a μR agonist, 30 min before the probe test spent a significantly reduced time in the target quadrant in the MWM task. Administration of EP stress before the probe test produced a reduction in the target quadrant time very similar to that in the morphine treated mice (Figure 2A). To address the underlying neuronal mechanisms of this morphine- or stress-elicited memory deficit, the effect of μR activation exogenously by systemic morphine injection or endogenously by EP stress on excitatory synaptic transmission and plasticity of the hippocampus were examined by recording the SC stimulation-evoked fEPSPs and PSs, respectively, of CA1 pyramidal neurons in anesthetized mice.

### Neither Stress Experience nor Exogenous Activation of μRs Alters Basal Excitatory Synaptic Transmission in the CA1 Region

Consistent with previous reports (Yang et al., 2006; Macdougall and Howland, 2013), there was no significant difference between stressed and unstressed mice in the input/output curves (Figure 2B) or PPR (Figure 2C) of fEPSP amplitudes, suggesting that EP stress does not affect SC-CA1 basal synaptic transmission. Furthermore, under anesthesia, the systemic injection of morphine (15 mg/kg, i.p.) to both unstressed and stressed mice had no significant effect on the fEPSP amplitude (Figure 2D). These results indicate that neither exogenous activation of μRs nor stress experience alter the basal excitatory synaptic transmission in the CA1 region.

### EP Stress Blunts GABAergic Feedforward Inhibition on Hippocampal CA1 Pyramidal Neurons by Activating μR_GABA_

**FIGURE 3:**
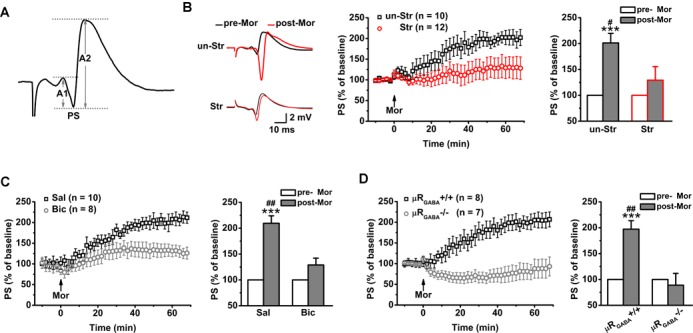
EP stress blocks the μR_GABA_-mediated reduction of GABAergic feedforward inhibition. The histograms in **(B–D)** show the average PS amplitude of the 5 min period immediately before and 55 min after morphine application, respectively. **(A)** The representative traces of evoked PSs. The PS amplitude was calculated using the formula (A1 + A2)/2. **(B)** The effects of morphine injection (15 mg/kg, i.p.) on the PS amplitude in unstressed and stressed wildtype mice. Treatment (morphine) *F*_(1_, _20)_ = 15.23, *p* = 0.001; group (stress) *F*_(1_, _20)_ = 4.64, *p* = 0.044; treatment × group *F*_(1_, _20)_ = 4.64, *p* = 0.044; R-M ANOVA. **(C)** The effects of morphine injection on the PS amplitude in unstressed wildtype mice pretreated with bicuculline or saline. Treatment (morphine) *F*_(1_, _16)_ = 29.72, *p* = 0.000; group (saline or bicuculline) *F*_(1_, _16)_ = 9.21, *p* = 0.008; treatment × group *F*_(1_, _16)_ = 9.21, *p* = 0.008; R-M ANOVA. **(D)** The effects of morphine injection on the PS amplitudes in unstressed μR_GABA_-/- mice and their littermates (μR_GABA_+/+). Treatment (morphine) *F*_(1_, _13)_ = 9.65, *p* = 0.008; genotype *F*_(1_, _13)_ = 15.26, *p* = 0.002; treatment × genotype *F*_(1_, _13)_ = 15.26, *p* = 0.002; R-M ANOVA. One symbol, *p* < 0.05; two symbols, *p* < 0.01; three symbols, *p* < 0.001; ^∗^paired Student’s *t*-test within-group; ^#^unpaired *t*-test between-group. un-Str, unstressed; Str, stressed; Sal, saline; Bic, bicuculline; Mor, morphine; pre-Mor, baseline before morphine application; post-Mor, 55 min after morphine.

In contrast to fEPSPs, which are less sensitive to μR activation, the evoked PS of CA1 pyramidal neurons in unstressed mice increased dramatically in amplitude after systemic injection of morphine (201.25 ± 18.43% of the pre-morphine level, measured 55 min after morphine application) in anesthetized mice (Figure 3B). This potentiation of the PS amplitude may reflect enhanced excitatory synaptic drive and/or increased intrinsic excitability of pyramidal neurons, and/or depressed GABAergic feedforward inhibition on CA1 pyramidal neurons. Given that activation of μRs does not directly modulate fEPSPs (Figure 2C) or the intrinsic excitability of pyramidal neurons (Siggins and Zieglgansberger, 1981), the potentiation of the PS amplitude by morphine application putatively originates in the attenuation of feedforward inhibition through activation of μR_GABA_. To address this possibility, we blocked GABAergic inhibition with bicuculline (3.0 mg/kg, i.p.), a GABA_A_ receptor-specific antagonist, 30 min prior to morphine administration in anesthetized mice. As expected, pretreatment with bicuculline significantly inhibited the morphine-induced potentiation of the PS amplitude compared to pretreatment with vehicle (saline) (Figure 3C). Furthermore, compared with μR_GABA_+/+ mice, μR_GABA_-/- mice showed a complete absence of the morphine-induced PS potentiation (Figure 3D). These results confirm that activation of μR_GABA_ reduces GABAergic neuron-mediated feedforward inhibition of PSs.

Intriguingly, EP stress also disrupted the morphine-induced enhancement of the PS amplitude (Figure 3B) in a manner comparable with that observed in unstressed mice that received bicuculline pre-treatment (Figure 3C) and unstressed μR_GABA_-/- mice (Figure 3D), showing that the capability of morphine to reduce feedforward GABAergic inhibition is blunted by acute stress. These results indicate that the endogenous activation of μRs during EP stress precludes the effect of exogenous agonists such as morphine.

### EP Stress Attenuates GABAergic Feedback Inhibition on Hippocampal CA1 Pyramidal Neurons via Activation of μR_GABA_

**FIGURE 4:**
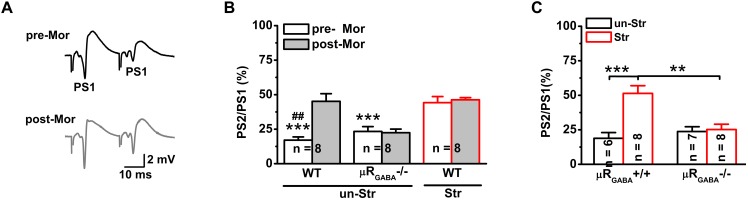
EP stress inhibits GABAergic feedback inhibition through activation of μR_GABA_. **(A)** Representative traces of paired-pulse PS with an IPI of 25 ms in unstressed wildtype mice pre- (upper) and post- (lower) morphine injections. **(B)** The effects of morphine on PS PPD. Treatment (morphine) *F*_(1_, _21)_ = 8.71, *p* = 0.008; group *F*_(2_, _21)_ = 26.93, *p* = 0.000; treatment × group *F*_(2_, _21)_ = 7.78, *p* = 0.003; R-M ANOVA; ^∗∗∗^*p* < 0.001 versus WT + Str, Bonferroni *post hoc* test between-group; ^##^*p* < 0.01, paired Student’s *t*-test within-group. **(C)** The effects of stress on the PS PPD in μR_GABA_-/- mice. Two-way ANOVA, *F*_(3_, _25)_ = 11.16, *p* = 0.000; treatment (stress) *F*_(1_, _25)_ = 14.27, *p* = 0.001; genotype *F_(_*_1_, _25)_ = 5.56, *p* = 0.026; treatment × genotype *F*_(1_, _25)_ = 11.97, *p* = 0.002. ^∗∗^*p* < 0.01 and ^∗∗∗^*p* < 0.001, Bonferroni *post hoc* test. pre-Mor, baseline before morphine application; post-Mor, 55 min after morphine; WT, wildtype; un-Str, unstressed; Str, stressed.

Next, we investigated the effects of stress on GABAergic neuron-mediated feedback inhibition, the other fundamental mechanism of regulating the timing of action potential generation by controlling the temporal summation of excitatory inputs. The paired-pulse depression (PPD, the second response is smaller than the first response) of PSs induced by short IPIs (<50 ms) reflects GABA_A_ receptor-mediated inhibitory effect on spike of pyramidal neurons and is widely used to evaluate GABAergic neuron-mediated feedback inhibition (Lupica and Dunwiddie, 1991; Sloviter, 1991; Uruno et al., 1995; Levkovitz and Segal, 1998; Wu and Leung, 2001; Zappone and Sloviter, 2004). Here, we used the PPR paradigm of PSs with an IPI of 25 ms. Unstressed mice exhibited a strong PPD of the PS PPR (PPR = 19.90 ± 3.51%), showing feedback inhibition of the GABAergic neurons innervating the pyramidal cell layer (Davies et al., 1990; Lupica and Dunwiddie, 1991; Karnup and Stelzer, 1999). Morphine injection (15 mg/kg, i.p.) significantly inhibited the PS PPD in unstressed mice (Figure 4A,B), indicating a blunting effect of morphine on feedback inhibition. The morphine injection did not cause a similar inhibition of the PS PPD in μR_GABA_-/- mice (pre-morphine versus post-morphine, *p* > 0.05, paired-Student’s *t*-test) (Figure 4B), indicating that the morphine-induced inhibition of the PS PPD depends on the activation of μRs expressed on GABAergic neurons.

Interestingly, behavioral stress also significantly inhibited the PS PPD (stress increased the PPR by 192.59 ± 41.15% compared to the level in unstressed wildtype mice) in a manner similar to morphine application (morphine increased the PPR by 224.06 ± 70.82% compared to the pre-morphine level) in unstressed mice. Furthermore, morphine injection was no longer able to inhibit the PS PPD in stressed mice (pre-morphine versus post-morphine, *p* > 0.05) (Figure 4B), indicating that the endogenous activation of μRs during EP stress could prevent the effect of morphine. The stress-inhibited PPD relies on μR_GABA_, while the PPD inhibition observed in stressed μR_GABA_+/+ littermates was completely abolished in stressed μR_GABA_-/- mice (Figure 4C).

Taken together, these data demonstrate that EP stress alters the properties of PSs without affecting fEPSPs *in vivo*, implying that stress preferentially suppresses GABAergic inhibitory transmission in the hippocampal CA1 region by endogenously activating μR_GABA_.

### EP Stress Facilitates LTD Induction via Activation of μR_GABA_

Previous studies have demonstrated that acute stress facilitates LTD induction in glutamatergic synapses in the CA1 region of the hippocampus (Xu et al., 1998; Wong et al., 2007), which is thought to be of the underlying mechanisms causing memory deficits after stress. Here, we asked whether the facilitated LTD at SC-CA1 synapses is associated with endogenous activation of μRs during stress.

**FIGURE 5:**
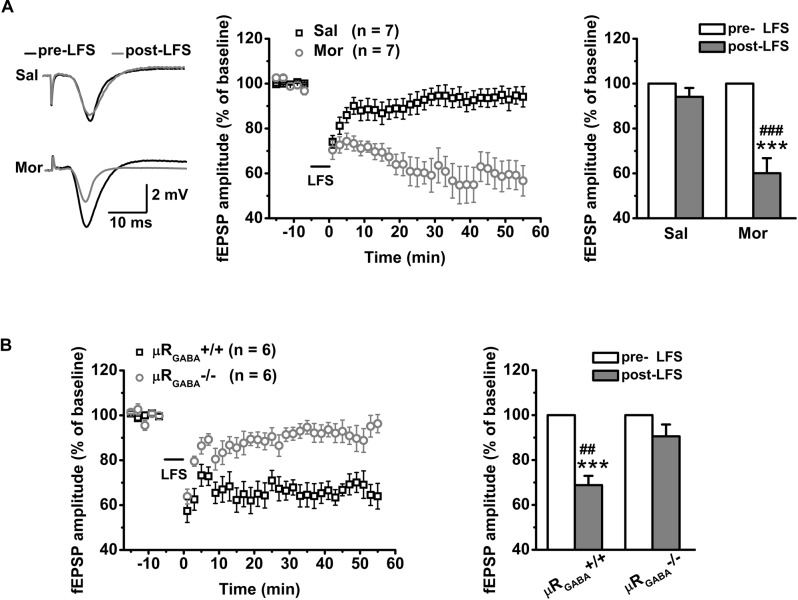
The effects of morphine pretreatment on LTD induction at hippocampal SC-CA1 synapses in adult wildtype and μR_GABA_-/- mice. The histogram in each figure shows a summary of fEPSP amplitude measurements pre- and post-morphine treatment. **(A)** Morphine but not saline administration 30 min before LFS facilitates LTD induction in wildtype mice. LFS (pre- and post-) *F*_(1_, _12)_ = 34.74, *p* = 0.000; group (saline or morphine) *F*_(1_, _12)_ = 19.15, *p* = 0.001; LFS × treatment *F_(_*_1_, _12)_ = 19.15, *p* = 0.001; R-M ANOVA. **(B)** Morphine facilitated LFS-LTD induction in μR_GABA_+/+ mice is absent in μR_GABA_-/- mice. LFS *F*_(1_, _10)_ = 36.78, *p* = 0.000; genotype *F*_(1_, _10)_ = 10.54, *p* = 0.009; LFS × genotype *F*_(1_, _10)_ = 10.54, *p* = 0.009; R-M ANOVA. Two symbols, *p* < 0.01; three symbols, *p* < 0.001; ^∗^paired Student’s *t*-test within-group; ^#^unpaired *t*-test between-group. Mor, morphine. Sal, saline.

For adult unstressed mice that received saline injection, the average fEPSP amplitude 45 min after LFS decreased slightly (decreased by 10.90 ± 5.06% compared to the pre-LFS level) but was not significantly different from the amplitude before LFS (*p* = 0.08, pre- versus post-LFS, paired Student’s *t*-test) (Figure 5A). In contrast, the application of morphine (15 mg/kg, i.p.) 30 min prior to LFS vigorously facilitated LTD expression (the average fEPSP amplitude decreased by 42.20 ± 6.59% compared to the pre-morphine level) in unstressed mice (Figure 5A). This facilitation of LFS-LTD by morphine pretreatment is dependent on μR_GABA_, as it was abolished completely by selective deletion of μR_GABA_ (*p* = 0.132, pre- versus post-LFS, paired Student’s *t-*test) (Figure 5B). Thus, the exogenous activation of μRs on GABAergic neurons can facilitate LTD induction on glutamatergic synapses in the hippocampal CA1 region of mice *in vivo*.

**FIGURE 6:**
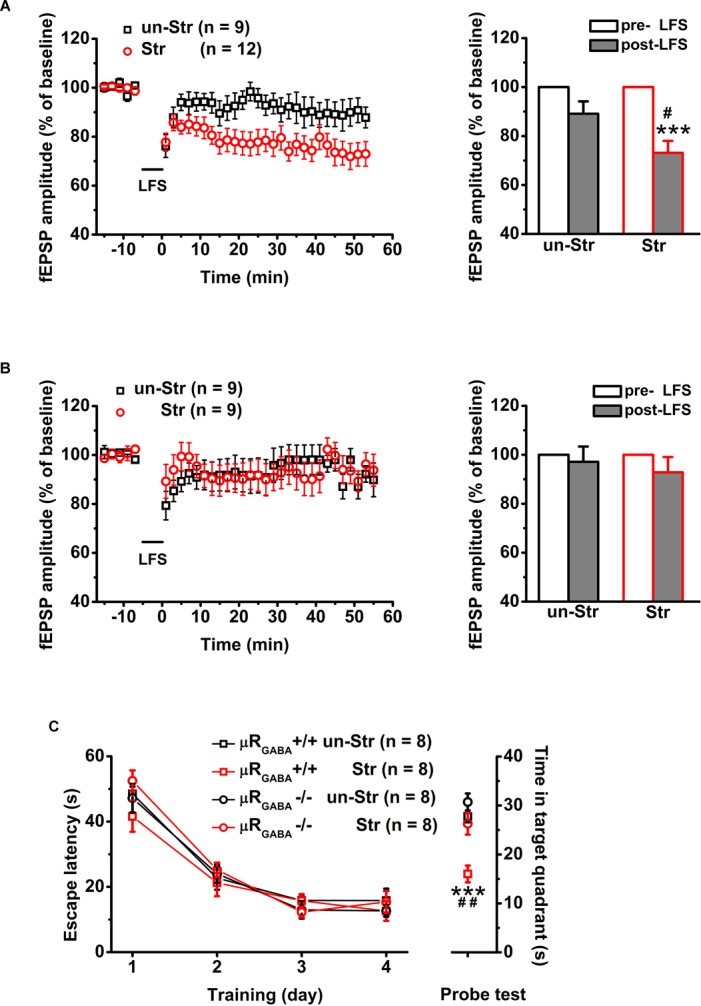
The facilitation of LTD induction and impairment of memory retrieval by EP stress depend on the activation of μR_GABA_. **(A)** LTD is induced by LSF in stressed wildtype mice. LFS (pre- and post-) *F*_(1_, _19)_ = 28.30, *p* = 0.000; group (stress) *F*_(1_, _19)_ = 4.81, *p* = 0.041; LFS × group *F*_(2_, _16)_ = 5.06, *p* = 0.037; R-M ANOVA; ^∗∗∗^*p* < 0.001, paired Student’s *t*-test within-group; ^#^*p* < 0.05, unpaired Student’s *t*-test between-group. **(B)** LFS fails to induce LTD in both stressed and unstressed μR_GABA_-/- mice. LFS *F*_(1_, _16)_ = 1.30, *p* = 0.271; group *F*_(1_, _16)_ = 0.24, *p* = 0.631; LFS × group *F*_(1_, _16)_ = 0.24, *p* = 0.631; R-M ANOVA. **(C)** The EP stress-induced impairment of memory retrieval in wildtype mice is reversed in μR_GABA_-/- mice. All groups exhibit a similar escape latency during MWM training days [group *F*_(3_, _28)_ = 0.82, *p* = 0.494; training day *F*_(3_, _84)_ = 115.86, *p* = 0.000; group × training day *F*_(9_, _84)_ = 0.77, *p* = 0.625; R-M ANOVA]. Time spent in target quadrant of test trial, genotype *F*_(1_, _28)_ = 14.07, *p* = 0.001; treatment (stress) *F*_(1_, _28)_ = 21.19, *p* = 0.000; genotype × treatment *F*_(1_, _28)_ = 4.33, *p* = 0.047; two-way ANOVA. ^∗∗∗^*p* < 0.001, versus un-stressed μR_GABA_+/+; ^##^*p* < 0.01, versus stressed μR_GABA_-/-; Bonferroni *post hoc* test. un-Str, unstressed; Str, stressed.

Next, we asked if acute stress facilitates LTD induction via endogenous activation of μRs. Consistent with previous studies (Xu et al., 1997; Wong et al., 2007), a robust LTD was observed in EP stressed mice (the fEPSP amplitude decreased by 26.88 ± 4.88% compared to the pre-LFS level) (Figure 6A), demonstrating that behavioral stress facilitated LTD induction. No significant LFS-LTD was observed in either stressed (*p* = 0.283, versus pre-LFS, paired Student’s *t*-test) or unstressed (*p* = 0.657, versus pre-LFS, paired Student’s *t*-test) μR_GABA_-/- mice (Figure 6B), indicating that EP stress-facilitated LTD induction also depends on the activation of μR_GABA_. These results suggest that acute stress endogenously activates μR_GABA_, and this activation is required for LTD facilitation.

Lastly, behavioral experiments revealed that the EP stress-induced reduction in time spent in the target quadrant in the WMW task (Figure 2A) was reversed in μR_GABA_-/- mice (Figure 6C).

## Discussion

**FIGURE 7:**
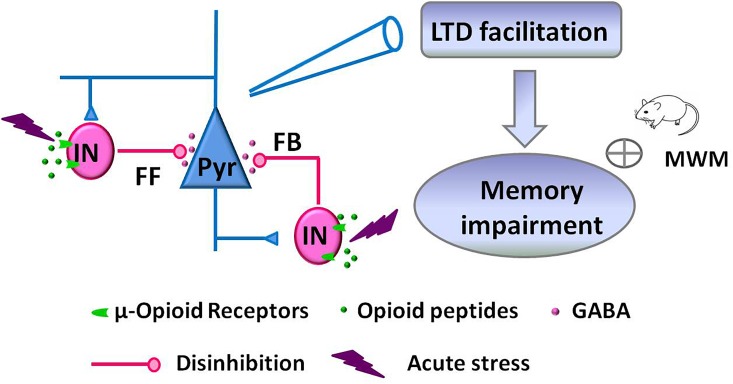
Schematic illustration showing the proposed mechanism of memory impairments induced by acute stress. EP stress exposure decreased GABA release from interneurons (IN) and attenuated GABAergic feedforward (FF) and feedback (FB) inhibition of CA1 pyramidal neurons (Pyr). These disinhibition effects facilitated LFS-induced LTD at SC-CA1 glutamatergic synapses, which leading to the memory impairments.

Facilitation of NMDAR-dependent LFS-LTD in the hippocampal CA1 region of the adult rodent brain is closely linked to stress-induced impairment of memory retrieval (Kim et al., 1996; Xu et al., 1997; Wong et al., 2007). This alteration of glutamatergic synaptic plasticity is mediated by the activation of glucocorticoid receptors, GluN2B-containing NMDAR, and protein synthesis (Kim et al., 1996; Xu et al., 1998; Joels et al., 2006). Here, we further demonstrate that acute stress alters GABAergic synaptic transmission via activation of hippocampal μR_GABA_, as supported by the following evidence: (1) EP stress attenuated both GABAergic feedforward and feedback inhibition of hippocampal CA1 pyramidal neurons in a μR_GABA_-dependent manner. (2) EP stress facilitated μR_GABA_-dependent LFS-LTD induction. Such stress-induced attenuation of hippocampal GABAergic signaling alters glutamatergic synaptic plasticity and contributes to the learning and memory impairments associated with stress (Figure 7).

The EOS has long been implicated in stress-related behaviors and physiological responses. On the one hand, stress induces an increase in enkephalin, dynorphin, and β-endorphin release in stress-related brain regions (such as the amygdala, hypothalamus, locus coeruleus, nucleus accumbens, hippocampus, etc.). These endogenous opioids activate opioid receptors (μ, δ, κ) to modulate the hypothalamic–pituitary–adrenal axis, autonomic nervous system, and behavioral responses during stress (Bali et al., 2015; Valentino and Van Bockstaele, 2015). In particular, the μR-mediated opioid signaling pathway plays a key role in stress coping and has anti-stress effects, such as reducing the risk of developing depression and attenuating stress-induced anxiety (Bali et al., 2015; Valentino and Van Bockstaele, 2015). On the other hand, the μR signaling pathway has also been shown to be involved in various cognitive tasks, including hippocampus-dependent memory processing (Fichna et al., 2007; Kitanaka et al., 2015; Kibaly et al., 2016). Peripheral or intra-hippocampal administration of naloxone or naltrexone (both μR antagonists) blocks the deficit in memory retrieval induced by acute stress or glucocorticoids (Rashidy-Pour et al., 2004; Sajadi et al., 2007; Colgin et al., 2009; Cao et al., 2015; Liu et al., 2016), suggesting that opioid receptors participate in stress-related memory deficits. The present results provide a reasonable explanation for these behavioral observations: acute stress alters GABAergic inhibition of hippocampal CA1 pyramidal neurons by endogenously activating μRs, thereby altering excitatory synaptic plasticity and ultimately impacting memory retrieval.

GABAergic interneurons are essential for maintaining a functional balance between excitatory and inhibitory neuronal activity throughout the brain. Their network activities regulate activity-dependent excitatory synaptic plasticity (Meredith et al., 2003) and control the oscillations of the hippocampal network, both of which are thought to be essential for memory performance (Csicsvari et al., 2003). Neural disinhibition of the hippocampus caused by dysfunction of GABAergic neurons has been implicated in memory deficits and psychiatric disorders (Marin, 2012). The selective reduction of functional inhibitory synapses or GABAergic depletion in the dorsal hippocampus impairs spatial learning and memory (Reichel et al., 2014). The μR is a vital modulator of the function of GABAergic interneurons. In the hippocampus, μRs are exclusively expressed on GABAergic inhibitory interneurons (Drake and Milner, 2002). Endogenous or exogenous activation of μRs can inhibit the firing rate and neurotransmitter release of the interneurons. GABAergic interneuron-mediated feedforward and feedback inhibition are two fundamental modes of regulating the timing of action potential generation by controlling the temporal summation of excitatory inputs (Pouille and Scanziani, 2001, 2004). We found that EP stress reduced the feedforward (Figure 3) and feedback inhibition (Figure 4) of hippocampal CA1 pyramidal neurons via activation of μR_GABA_ on GABAergic neurons. The PS amplitude that reflects the recruitment of spiking neurons depends both on excitatory and inhibitory synaptic inputs. The previous study showed that blockade of GABAergic transmission with bicuculline caused a significant increase in the PS amplitude without affecting the fEPSP (Levkovitz and Segal, 1998). The present study revealed that EP stress altered the PS without affecting the basal fEPSP (Figure 2). These results suggest that the inhibitory input rather than the excitatory input to pyramidal neurons is impacted by EP stress, and such depression of inhibitory input disinhibits pyramidal neurons. The reduction in feedforward and feedback inhibition by activation of the μR_GABA_ located in the stratum oriens and the stratum lacunosum moleculare not only modulates hippocampal network oscillations (Lupica and Dunwiddie, 1991) but also alters glutamatergic synaptic plasticity (Wagner et al., 2001), both of which are associated with hippocampus-based memory encoding/retrieval (Colgin et al., 2009; Ruediger et al., 2011).

In brain slices, activating μRs exogenously using a selective agonist or endogenously by enhancing the release of opioids facilitates 1-Hz LFS-induced LTD at SC-CA1 synapses through modulation of feedforward inhibition (Wagner et al., 2001). Another report showed that exogenously activating μRs also facilitated a 3-Hz LFS-LTD at similar synapses, but this effect was independent of GABA-mediated inhibitory neurotransmission (Tian et al., 2015). Although both forms of the SC-CA1 LTD in vitro require NMDAR activation (Cui et al., 2013), the difference between these two studies raises questions about the functional site of μRs in LTD induction. For example, the activation of μRs in astrocytes cannot be excluded, and μRs are highly expressed on astrocytes in the CA1 hippocampus (Nam et al., 2018). However, the present results show that the facilitation of 3-Hz LFS-LTD *in vivo* depends on the activation of μR_GABA_, which confirms that GABA-mediated inhibitory neurotransmission is required. Although the distinct mechanisms underlying the effects of GABAergic transmission on glutamatergic synaptic plasticity remain speculative, there are several possible ways to generate GABAergic transmission-mediated LTD facilitation: (1) the reduction in postsynaptic GABA_A_ receptor-mediated GABA response depolarizes pyramidal neurons and thus promotes LTD induction by lowering the threshold for NMDAR activation (removes shunting and hyperpolarizing effects) (Wagner and Alger, 1995); (2) the reduction in postsynaptic GABA_A_ receptor activation also enhances cAMP/PKA signaling, which subsequently leads to LTD (cAMP-mediated LTD) (Yu et al., 2003); (3) reduced activation of GABA_B_ receptors at presynaptic excitatory terminals, whose function is to inhibit synaptic transmission via G protein-mediated modulation of presynaptic Ca^2+^ channels, may also facilitate LTD induction. In addition to the CA1 area, μR-mediated facilitation of LTD induction were also reported at excitatory synapses in the dorsal striatum (Atwood et al., 2014) and paraventricular nucleus of the hypothalamus (Wamsteeker Cusulin et al., 2013), indicating μR-mediated long-term plasticity exists widely in emotion- and cognition-related brain regions thereby modulating the relevant behaviors.

Notably, exogenous activation of μRs facilitates LTD induction (Wagner et al., 2001) but suppresses LTP induction (Tian et al., 2015) in SC-CA1 synapses in a manner very similar to the effects of acute stress (Kim et al., 1996). In addition, acute stress exposure precludes the effect of μR activation on synaptic plasticity (Yang et al., 2004). These data indicate that the μ-opioid system is involved in mediating the effects of stress on synaptic plasticity. Our data demonstrating the absence of acute stress-facilitated LTD in μR_GABA_-/- mice strongly supports that μRs on GABAergic interneurons play a crucial role in stress-related modulation of synaptic plasticity. Consistent with this finding, recent studies have shown that exposure to stress can alter the GABAergic function via changes in GABA release and the expression of specific GABA_A_ receptor subunits (Maggio and Segal, 2009); such changes interrupt the normal balance of inhibition and excitation in memory- and emotion-related circuits in the brain (Tzanoulinou et al., 2014; Lee et al., 2016; Hadad-Ophir et al., 2017). In addition to the spatial reference memory observed presently, the opioid receptor-mediated pathway is reportedly involved in stress-altered other hippocampus-dependent learning and memory, such as the inhibitory avoidance task (Rashidy-Pour et al., 2004) and recognition memory task (Liu et al., 2016), implying they might share the similar mechanism. The overall effects of this μR-mediated specific GABAergic pathway on the modulation of synaptic plasticity at excitatory synapses remain to be elucidated, including phasic versus tonic inhibitory currents, feedforward versus feedback inhibition, and other aspects. However, the present work provides novel insight into the cellular mechanisms underlying stress-induced memory deficits.

## Author Contributions

Z-QL and M-MS designed the research, drafted the manuscript, and supervised the project. K-MF performed the behavioral experiments, analyzed data, and prepared figures. L-JQ collected the electrophysiological data. NM and Y-ND performed *in situ* hybridization. Z-QQ and C-LW verified the genotype of mutant mice. WR and JH provided revisions to the overall the manuscript. All authors agree to be accountable for the content of the work.

## Conflict of Interest Statement

The authors declare that the research was conducted in the absence of any commercial or financial relationships that could be construed as a potential conflict of interest.

## Abbreviations

EOS: the endogenous opioid system
EP: elevated platform
fEPSPs: field excitatory postsynaptic potentials
IPI: interpulse interval
LFS: low-frequency stimulation
LTD: long-term depression
μRs: μ-opioid receptors
μR_GABA_: μRs on GABAergic neurons
NMDAR: N-methyl-D-aspartate receptor
PPD: paired-pulse depression
PPR: paired-pulse ratio
PSs: population spikes
R-M ANOVA: repeated-measures analysis of variance
SC: Schaffer collaterals/commissural
